# Supplementary material for: Patterns of Intron Gain and Loss in Fungi
Source: PLoS Biol. 2004 Nov 30;2(12):e422. doi: 10.1371/journal.pbio.0020422 (PMC532390; doi:10.1371/journal.pbio.0020422)
Supplement: Table S1 — Also available at http://genes.mit.edu/NielsenEtAl/. (4.3 MB ZIP). [file pbio.0020422.st001.zip › NielsenEtAl/html/241.html]

AN7659.1.NCU01160.1.MG07333.1.FG10087.1


```
 CLUSTAL W (1.82) Multiple Sequence Alignments - Introns Inserted


Sequence 1: NCU01160.1	483 aa
Sequence 2: MG07333.1	504 aa
Sequence 3: FG10087.1	488 aa
Sequence 4: AN7659.1	477 aa
Alignment Length: 522 aa
Number Identitical Residues: 213 aa
Alignment Score (without introns) 10630


MG07333.1 	MQTYNASSQQQANSWAFNPWPPAQNQAMSDLASRITAPAAATPATETDAPAA~ASST--L
NCU01160.1	---------------------------MADLASRITKPDEAPAAAPEAAPVS~APASEEP
FG10087.1 	---------------------------MADLASRITKPDEVAAETPAETPAE~TAPAASG
AN7659.1  	------------------------------MAS--DAPTGGSLADRISNPAE1TTEP-VA
          	                              :**    *   .       *.  :. .   

MG07333.1 	AVPDGAADGANS--SGLQESNYDVEVQLGDP--DTDSPLSSISSFSELGL2PQGIIDGLL
NCU01160.1	KAPENETSIEESQ-SNLVKNEYEVEIKLSDLQNDTESPLYSVSSFDELGL~PEAVNRGLL
FG10087.1 	ELGQADGNIEDLGGSGLQEPEWDVEVSLSELQNNEATPFHSATTWQDLGL2REDLLKGLL
AN7659.1  	DKAQLDGAASNQGGSDLAEPEYNVEVKLSDLQADPNNPLYSVKNFEDLGL2DPRILQGLS
          	   :      :  .*.* : :::**:.*.: . :  .*: * ..:.:***    :  ** 

MG07333.1 	AMNFKKPSKIQARALPLMLSNPPRNMIAQSQSGTGKTGAFVVTILSRVDFNQ---PNQPQ
NCU01160.1	AINFKKPSKVQEKCLPLMLSDPPRNMIAQSQSGTGKTAAFVLTVLSRIDLSK---PHQPQ
FG10087.1 	SLNFLKPSKVQGKSLPLMLSDPPRNMLAQSQSGTGKTAAFVTAILSRVDFSK---PDQPQ
AN7659.1  	AMNFRKPSKIQERALPLLMGNPPKNLVGQSQSGTGKTAAFVLNILSRLDLSSEQAQKTPQ
          	::** ****:* :.***::.:**:*::.*********.***  :***:*:....: . **

MG07333.1 	ALALAPSRELARQIQSVIQSIGQFCTGLVVDAAIPG-AISRETGVKANVVVGTPGTVMDL
NCU01160.1	ALLLAPSRELARQIQTVVQTIGQFCENLIVEAAIPG-AISRETGVRGSVVVGTPGTVMDL
FG10087.1 	ALALAPSRELARQIEGVINAIGRFVENKKVAAAIPG-VLPRGEPVRASVIVGTPGTVMDI
AN7659.1  	ALILAPTRELARQIVGVIQVMGKFLDGLHIGTAVPADTNARPTRMEASVVVGTPGTVMDM
          	** ***:*******  *:: :*:*  .  : :*:*... .*   :...*:*********:

MG07333.1 	IRRRQFDVSQLKLLVVDEADNMLDQQGLGEQCVRVKN2MLPKTIQTLLFSATFPDHVKSY
NCU01160.1	VKRRQFDISQLKVLVIDEADNMLDQQGLGDQCVRVKN2MLPKTIQILLFSATFPDKVLRF
FG10087.1 	IRRRQLDISQLRVLVLDEADNMLDQQGLGDQCLKVKN2MLPKEIQVLLFSATFPENVMKY
AN7659.1  	IKKRIMVAAKLKVIVLDEADNMLDQQGLGDQCIRVKA2LLPRDIQVVLFSATFPAHVHQY
          	:::* :  ::*:::*:*************:**::**  :**: ** :******* :*  :

MG07333.1 	AEKFAPQANQMKLRQQELTVKGISQMYMDCPSLKEKYEVLCKLYGLMTIGSSVIFVK0TR
NCU01160.1	AERFAPNANQMKLKHKELTVKGISQMFMDCPTEKDKYDILCKLYGLMTIGSSVIFVR0TR
FG10087.1 	AGKFAPNAHSLKLQRSELTVKGISQMFIDCPDDNMKYDILCKLYGLMTIGQSVIFVK0TR
AN7659.1  	ASKFAPAANELTLQHEELTVEGIKQLYLDCASEEDKYRTLVQLYGLLTVGSSIIFVK0TR
          	* :*** *:.:.*::.****:**.*:::**.  : **  * :****:*:*.*:***: **

MG07333.1 	ESADEIQRRMEADGHKVSALHGAFQGQERDQLLDDFRSGKSKVLITTNVLARGIDVSSVS
NCU01160.1	ETANEIQKRMEADGHKVSALHGAYEGQSRDVLLDEFRSGRSKVLITTNVLARGIDVSSVS
FG10087.1 	DSASEIERRMVADGHKVSALHAAFDGAERDNLLTKFRQGENKVLITTNVLARGIDVSSVS
AN7659.1  	ASAVEIERRMVAEGHTVASLTGGIEGSQRDQIIDQFRAGHAKVLITTNVLARGIDVSTVS
          	 :* **::** *:**.*::* .. :* .** :: .** *. ****************:**

MG07333.1 	MVINY~DIPMKGPGDQSP---DAETYLHRIGRTGRFGRVGVSISFVHDRKSFTALSSIAE
NCU01160.1	MVINY~DIPMKGPGEREP---DAETYLHRIGRTGRFGRVGVSISFVHDRRSFEALSQIAQ
FG10087.1 	MVINY~DIPMKGRGDTEP---DAETYLHRIGRTGRFGRVGVSISFVYDKKSFDALSKIAE
AN7659.1  	MVINY0DIPELHQPPNRPRQADFQTYLHRIGRTGRFGRVGVSISFVSNREEWEMLNQIQK
          	***** ***        * .:* :********************** ::..:  *..* :

MG07333.1 	HYGIDLIQLSPDDWDDTEVKVQDVIKSSRAKPDYAPTQEKAA-----
NCU01160.1	FYGIDLIQLNPNDLDDTERKVQEVIKSSRAQAEYVPSATDSAV----
FG10087.1 	MYGIDLVKLDTEDWDEAEERVKEVIKKNRAQASYAPSATEPKAAAGA
AN7659.1  	YFNTDIQRIDTKDWDEVEDIIKKTIKNTRAQAGFR------------
          	 :. *: ::...* *:.*  ::..**..**:. :
```
